# High Glucose-Induced Increased Expression of Endothelin-1 in Human Endothelial Cells Is Mediated by Activated CCAAT/Enhancer-Binding Proteins

**DOI:** 10.1371/journal.pone.0084170

**Published:** 2013-12-23

**Authors:** Simona-Adriana Manea, Andra Todirita, Adrian Manea

**Affiliations:** Institute of Cellular Biology and Pathology ‘Nicolae Simionescu’ of the Romanian Academy, Bucharest, Romania; Institute of Cellular Biology and Pathology ‘Nicolae Simionescu’ of the Romanian Academy, Bucharest, Romania; Harvard Medical School, United States of America

## Abstract

High glucose-induced endothelial dysfunction is partially mediated by the down-stream pathophysiological effects triggered by increased expression of endothelin-1 (ET-1). The molecular control mechanisms of ET-1 synthesis are yet to be discovered. Members of the CCAAT/enhancer-binding proteins (C/EBP) family are important regulators of key metabolic processes, cellular differentiation and proinflammatory genes. In this study, we aimed at elucidating the role of C/EBP in mediating the high glucose effect on ET-1 expression in human endothelial cells (EC). Human umbilical vein cells (EAhy926) and primary cultures of human aortic EC were exposed to high levels of glucose (16.5–25 mM). Real-time PCR, Western blot, enzyme-linked immunosorbent assay, ET-1 promoter-luciferase reporter analysis, and chromatin immunoprecipitation assays were employed to investigate ET-1 regulation. High glucose activated C/EBPα, C/EBPβ, and C/EBPδ in a dose-dependent manner. It also promoted significant increases in ET-1 gene and peptide expression. Chemical inhibition of JNK, p38MAPK and ERK1/2 diminished significantly the high glucose-induced nuclear translocation of C/EBP and ET-1 expression. Silencing of C/EBPα, C/EBPβ or C/EBPδ greatly reduced the high glucose-induced upregulation of ET-1 mRNA, pre-pro-ET-1, and ET-1 secretion. The expression of various C/EBP isoforms was selectively downregulated by siRNA-mediated gene silencing. *In silico* analysis indicated the existence of typical C/EBP elements within human ET-1 gene promoter. Transient overexpression of C/EBPα, C/EBPβ or C/EBPδ upregulated the luciferase level controlled by the ET-1 gene promoter. The direct interaction of C/EBPα, C/EBPβ or C/EBPδ proteins with the ET-1 promoter in high glucose-exposed EC was confirmed by chromatin immunoprecipitation assay. High glucose-induced ET-1 expression is mediated through multiple mechanisms. We present evidence that members of the C/EBP proinflammatory transcription factors are important regulators of ET-1 in high glucose-exposed human endothelial cells. High glucose-induced activation of C/EBP-related signaling pathways may induce excessive ET-1 synthesis, thus promoting vasoconstriction and dysfunction of the vascular wall cells in diabetes.

## Introduction

Hyperglycemia, the primary clinical manifestation of diabetes, contributes to diabetic complications [1] by inducing vascular inflammation, oxidative stress, impaired vascular relaxation, changing vascular cell metabolism, altering the vascular matrix molecules, and circulating proteins/lipoproteins. [2]–[4] Nevertheless, the precise mechanisms by which hyperglycemia induce pathological outcomes and the molecular nature of its down-stream effectors is still a debatable issue.

Convincing evidence exists that the endothelin system plays an important role in the pathophysiology of diabetes-associated cardiovascular diseases. [5] The endothelin system comprises biological active peptides called endothelins, endothelin converting enzymes, and specific cellular receptors. [6]–[8] Endothelins regulate important physiological processes including vascular tonus [9], cellular growth and proliferation. [10] However, in pathological conditions such as diabetes mellitus, dysregulation of the endothelin system, characterized by enhanced expression, activity or responsiveness of different constituents contributes to dysfunction of the vascular cells. [11], [12]

Hyperglycemia-induced vascular deleterious effects are partially mediated by the endothelin-1 (ET-1). Increased synthesis of ET-1, the main effector of the endothelin system, induces vasoconstriction, dysfunction of endothelial cells (EC), phenotypic alteration of smooth muscle cells, vascular remodeling, inflammation and oxidative stress. [13] Multiple mitogenic signaling pathways [(e.g., mitogen-activated protein kinases (MAPK), Janus kinase (Jak)] and pro-inflammatory transcription factors such as nuclear factor kB (NF-kB), activator protein 1 (AP-1), and members of the signal transducer and activator of transcription (STAT) family have been implicated in the regulation of ET-1 expression. [14]–[16] However, the precise molecular pathways responsible for increased ET-1 level in diabetes are not totally deciphered.

Evidence is accumulating that the basic-leucine zipper transcription factor family, CCAAT/enhancer-binding proteins (C/EBP), plays a major role in cellular differentiation and function. [17] The C/EBP family consists of six members (C/EBP-α, -γ, -β, -δ, -ε, -ξ) each with a distinct cell and tissue distribution. Upon activation, C/EBPs form homo- or heterodimers and interact with the cytidine-cytidine-adenosine-adenosine-thymidine box motif in the enhancers and promoters of target genes, and regulate important biological activities such as metabolism, cellular proliferation, growth, and differentiation. [18]

Various members of the C/EBP family, namely C/EBPα, -β, and –δ have been proved to regulate the expression of many cytokines, chemokines, growth factors, acute phase proteins, and immunoglobulins. [17], [19] Still, the precise function of C/EBPs in the cardiovascular system is still a matter of debate.

Based on the fact that C/EBPs transduce the effects of numerous pro-inflammatory and growth-related stimuli, we examined the role of C/EBP in mediating high glucose-induced ET-1 level in cultured EC. We provide evidence that C/EBPα, C/EBPβ and C/EBPδ are activated by high glucose and that MAPK signaling, and C/EBPα, -β, and –δ isoforms are coordinately involved in the regulation of ET-1 expression in high glucose-exposed endothelial cells.

## Materials and Methods

### Materials

General chemicals and reagents, antibodies, siRNA, and molecular biology kits were derived from Sigma-Aldrich (Germany), Santa Cruz Biotechnology (USA), Invitrogen (Austria), Qiagen (Germany), R&D Systems (Austria). The enzyme-linked immunosorbent assay (ELISA)-based endothelin-1 detection kit was obtained from Biomedica (Austria). The pGL2 basic reporter vector carrying the human ET-1 gene promoter was kindly provided by Prof. Dr. Angelika Bierhaus (University Heidelberg, Germany). The C/EBPα, C/EBPβ and C/EBPδ expression vectors were from Thermo/OpenBiosystems, β-galactosidase from Promega (Germany), and the pC/EBP-luc control plasmid from Stratagene (Germany).

### Cell culture

Human EC line EAhy926 was purchased from the American Type Culture Collection (USA). The cells were cultured in Dulbecco's modified Eagle's Medium (DMEM) with 5.5 mM glucose, containing essential and non-essential amino acids (in mg/l: 8.9 L-alanine, 13.3 aspartic acid, 15 L-asparagine, 14.7 monosodium glutamate, 11.5 proline), sodium selenite (0.02 mg/l), ascorbic acid (10 mg/l), 10% fetal bovine serum (FBS; v/v), and antibiotics (100 units/ml penicillin, 100 µg/ml streptomycin, 50 µg/ml neomycin).

Confluent quiescent cells cultured for 24 h in serum-free DMEM (DMEM, 5.5 mM glucose) were further exposed for additional 24 h to DMEM comprising 5.5 or 25 mM glucose in the absence or presence of selective MAPK pharmacological inhibitors or specific siRNA for C/EBPα, C/EBPβ or C/EBP/δ. Optimal concentrations of inhibitors were established in preliminary dose-response experiments: 10 µM SP600125 (JNK), 10 µM SB203580 (p38MAPK), and 1 µM U0126 (ERK1/2).

In some experiments, commercial available primary cultures of adult human aortic EC (Lonza, Switzerland) were used to confirm that data obtained on EC line. The cells were grown to confluency according to the supplier's recommendations, and treated as described above.

### Real-Time PCR

The total cellular RNA was isolated from cultured EC and the first-strand cDNA synthesis was done employing M-MLV reverse transcriptase (Invitrogen). The quantification of ET-1 mRNA was done by amplification of cDNA using a real-time thermocycler (LightCycler®480 II, Roche, Switzerland) and SYBR™ Green I probe. The GeneBank™ accession number and the oligonucleotide primer sequences used in real time PCR reactions were: ET-1 (NM_001955) forward: 5′-CTTTGAGGGACCTGAAGCTG-3′, and reverse: 5′- AGTTCTTTTCCTGCTTGGCA-3′; GAPDH (NM_002046) forward: 5′-ACCACAGTCCATGCCATCAC-3′, and reverse: 5′-TCCACCACCCTGTTGCTGTA-3′; β-Actin (NM_001101) forward: 5′-CTGGCACCCAGCACAATG-3′, and reverse 5′-GCCGATCCACACGGAGTACT-3′. The optimal amplification settings were 0.2 µM of primers, 2.5 mM MgCl_2_, annealing at 60°C and extension at 72°C for 40 cycles. To validate the results, the expression levels were standardized by 2 housekeeping genes, GAPDH and β-Actin. [20] The mRNA quantification was done by means of the comparative C_T_ method and expressed as arbitrary units. [21]

### Western blot

*Total cellular extracts* – Cultured cells were washed in ice-cold PBS pH 7.4 before lysis in 2×Laemmli's electrophoresis sample buffer and boiled for 20 minutes.

*The isolation of nuclear fraction* was performed by using reagents and protocols from Santa Cruz Biotechnology. Briefly, the cells were wash cells twice with PBS pH 7.4 at room temperature and resuspended for 30 minutes with gentle mixing in ice-cold lysis buffer containing 5mM PIPES pH 8.0, 85 mM KCl, 0.5% NP-40 and protease inhibitor cocktail. The nuclear extract was collected by microcentrifugation at 2,000 RPM, 5 minutes, wash once with PBS pH 7.4, resuspended in 2×Laemmli's electrophoresis sample buffer as indicated above. The concentration of proteins was determined by Amido Black method. [22]

Equal amounts of protein (50 µg) were run on 12% (pre-pro-ET-1) or 10% (C/EBPα, C/EBPβ, C/EBPδ) SDS-PAGE and electroblotted onto nitrocellulose membranes. The membranes were exposed to blocking reagent TBS Blotto A (sc-2333), and then incubated overnight at 4°C with the primary antibodies against ET-1 (goat polyclonal, sc-21625), C/EBPα (goat polyclonal, sc-9314), C/EBPβ (rabbit polyclonal, sc-150), C/EBPδ (rabbit polyclonal, sc-636), Histone H1 (goat polyclonal, sc-34464), or β-Actin (mouse monoclonal, sc-47778), followed by horseradish peroxidase (HRP)-conjugated secondary antibodies. The protein bands were detected using chemiluminescence substrate solution (Pierce, Germany) and images were taken with a digital detection system (ImageQuant LAS 4000, Fujifilm, Japan). The quantification (TotalLab™) of the pre-pro-ET-1, C/EBPα, C/EBPβ, and C/EBPδ protein levels was determined by normalization to β-Actin (pre-pro-ET-1 and C/EBPs) or Histone H1 (C/EBPs) proteins and expressed as arbitrary units.

### Enzyme-linked immunosorbent assay

The level of ET-1 peptide was measured in the culture medium from EC exposed for 24 h to various concentrations of glucose (5.5 mM to 25 mM) in the absence/presence of siRNA directed to specific C/EBP isoforms. The analysis was performed with an ELISA kit according to the manufacturer's protocol (Biomedica).

### Transient transfection and luciferase assay

Exponentially growing cells were seeded 24 h prior transfection at 1.0×10^5^ cells/well (≈80% confluence) into 12-well tissue culture plates. Transient transfection was done as previously indicated [23] using Superfect™ reagent (Qiagen), DMEM supplemented with 10% FBS (v/v), 1 µg of luciferase construct (pET-luc, pC/EBP-luc), 0.1 µg pSV-β-galactosidase vector, 0.3 µg of expression vector (i.e., C/EBPα, C/EBPβ, C/EBPδ) or their corresponding empty vectors controls. The DNA/Superfect ratio was 1: 7.5 (wt/wt). The promoter activity was calculated from the ratio of firefly luciferase to β-galactosidase levels and expressed as arbitrary units.

### Transfection of siRNA

Twenty-four hours before siRNA transfection, exponentially growing EC were seeded at 4×10^5^ cells (≈50% confluence) in tissue culture plates (Ø 60 mm). C/EBPα (sc-37047), C/EBPβ (sc-29229), C/EBPδ (sc-37722), control (sc-37007) or fluorescein isothiocyanate (FITC)-labeled control (sc-36869) siRNA (20 nM) (Santa Cruz Biotechnology) were transfected into EC using Hiperfect™ reagent according to the manufacturer's protocol (Qiagen). To dislocate aggregates formed during lyophilization procedure, siRNA was incubated at 90°C for 1 minute and then at 37°C for 60 minutes before transfection procedure. [24] The transfection effectiveness into the cultured EC was assessed by fluorescence microscopy and the silencing efficiency was evaluated by Western blot, 48 hours after siRNA transfection.

### Chromatin immunoprecipitation assay

Chromatin immunoprecipitation (ChIP) assay was done using antibodies, reagents and protocols from Santa Cruz Biotechnology. Antibodies against C/EBPα (goat polyclonal, sc-9314), C/EBPβ (rabbit polyclonal, sc-150), C/EBPδ (rabbit polyclonal, sc-636) were used. PCR was performed with primers sets for the ET-1 gene promoter flanking the predicted C/EBP sites. A DNA fragment derived from the human p21 gene promoter was amplified from immunoprecipitated chromatin using primers from R&D Systems and served as positive control. ‘No-antibody’, IgG, and negative controls from genomic regions which do not contain predicted C/EBP elements (a DNA fragment derived from human c-Myc gene promoter) were employed. The specificity of PCR products was analyzed by gel electrophoresis and melting curve. Input DNA was amplified for each sample in parallel experiments.

### Statistical analysis

Data obtained from at least 3 independent experiments performed in duplicate were expressed as means ± standard deviation. Statistical analysis was performed by one-way ANOVA test; *P*<0.05 was considered statistically significant (*P<0.05, **P<0.01, ***P<0.001).

## Results

### High glucose up-regulates ET-1 gene and protein expression

**Figure 1:**
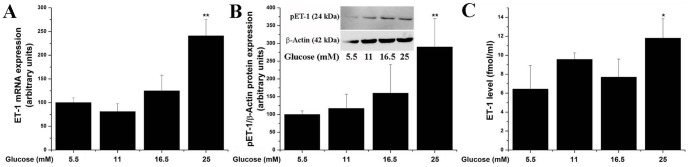
High glucose induces (A) ET-1 mRNA, (B) pre-pro-ET-1 expression, and (C) ET-1 peptide secretion. Cultured quiescent EC were exposed to the indicated concentrations of glucose for 24 h. The ET-1 mRNA was quantified by real-time PCR, pre-pro-ET-1 was assessed by Western Blot, and the ET-1 peptide released in the culture medium was determined by ELISA. n = 4, *P<0.05, **P<0.01. *P*-values were taken in relation to the 5.5 mM glucose-exposed cells.

To investigate the regulatory effect of high glucose on ET-1 expression, dose-dependent experiments were done in quiescent EC line exposed (24 h) to increasing concentrations of glucose (5.5–25 mM). As depicted in Figure 1, maximal effect of high glucose induced-upregulation of ET-1 mRNA, pre-pro-ET-1 and ET-1 peptide release was detected for 25 mM glucose. Similar findings were obtained in primary culture of human aortic EC (data not shown).

### High glucose induces C/EBP activation via MAPK signaling

**Figure 2:**
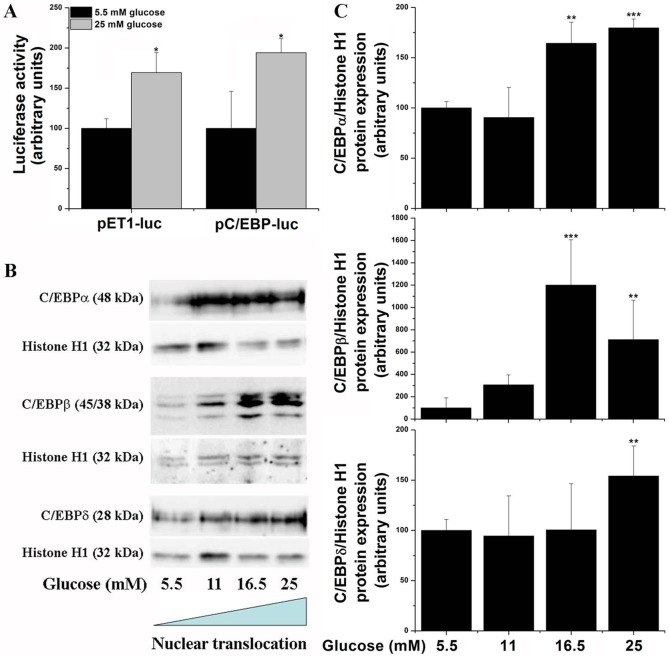
High glucose induces ET-1 gene transcription and activates C/EBPs. (A) Cells were transfected with either pET1-luc or pC/EBP-luc control plasmids as described under “Materials and Methods” in 5.5 mM-containing medium. Twenty-four hours after transfection cells were exposed for an additional 24 h to either 5.5 mM or 25 mM glucose. (B) Representative immunoblots depicting nuclear translocation of C/EBPα, C/EBPβ, and C/EBPδ in response to increasing concentrations of glucose (5.5 to 25 mM). (C) Densitometry analysis of C/EBP transcription factors activation. n = 3, *P<0.05. *P*-values were taken in relation to the 5.5 mM glucose-exposed cells.

Various synergistic approaches were employed to explore the potential of high glucose to activate C/EBP transcription factors. To search for the role of high glucose in activating C/EBP-associated signaling pathways in EC, transfection experiments were performed using pET1-luc and pC/EBP-luc, a control plasmid carrying three repetitive and highly conserved C/EBP elements up-stream to the luciferase reporter gene. EC were transiently transfected with indicated luciferase constructs in 5.5 mM glucose-containing medium, and 24 h after transfection (to achieve the baseline luciferase level) the EC were further exposed for additional 24 h to either normal (5.5 mM) or high (25 mM) glucose concentration. Transactivation assays showed that high glucose induced a significant up-regulation of the luciferase level directed the promoter of ET-1 gene, a process that was associated with the activation of C/EBP transcriptional responses (Figure 2A).

To further test this hypothesis, high glucose induced C/EBP activation was assessed by means of transcription factor nuclear translocations. As shown in Figure 2B and 2C, high glucose induced significant, but dissimilar activations of C/EBPα, C/EBPβ, and C/EBPδ.

**Figure 3:**
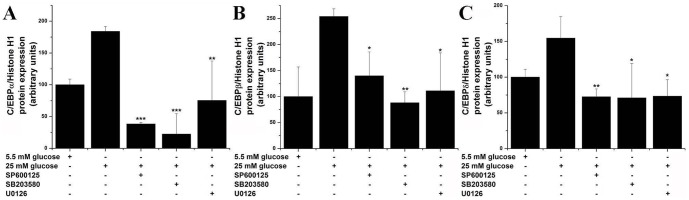
High glucose-induced C/EBP activation is mediated by MAPK. Activation of (A) C/EBPα, (B) C/EBPβ, and (C) C/EBPδ was investigated by means of nuclear translocation as indicated under “Materials and Methods”. Quiescent EC were exposed (24 h) to either 5.5 mM or 25 mM glucose in the absence/presence of vehicle (DMSO) or MAPK pharmacological inhibitors (10 µM SP600125, 10 µM SB203580, 1 µM U0126). n = 3, *P<0.05, **P<0.01, ***P<0.001. *P*-values were taken in relation to the 25 mM glucose-exposed cells.

**Figure 4:**
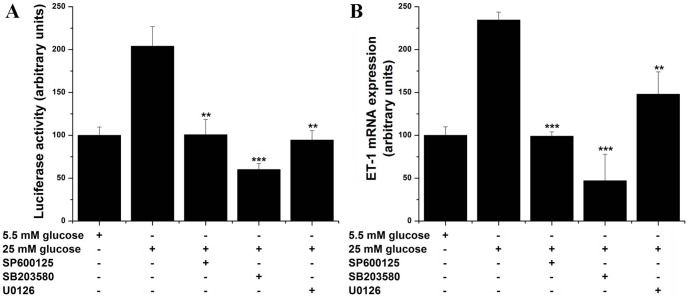
Inhibition of MAPK signaling blocks the high glucose-induced up-regulation of ET-1 transcription and mRNA expression. (A) Cultured cells were transfected with pET1-luc construct in 5.5 mM-containing medium. Twenty-four hours after transfection cells were exposed for an additional 24 h to either 5.5 mM or 25 mM glucose in the absence/presence of MAPK inhibitors (10 µM SP600125, 10 µM SB203580, 1 µM U0126). (B) Quiescent EC were exposed (24 h) to 5.5- or 25 mM glucose in the absence/presence of the indicated MAPK inhibitors and the mRNA was quantified by real-time PCR. n = 3, **P<0.01, ***P<0.001. *P*-values were taken in relation to the 25 mM glucose-exposed cells.

To gain additional mechanistically insights into the C/EBP activation in response to high glucose, various MAPK pharmacological inhibitors were used. Quiescent EC were exposed to either 5.5 mM or 25 mM glucose in the absence or presence of 10 µM SP600125 (JNK inhibitor), 10 µM SB203580 (p38MAPK inhibitor) or 1 µM U0126 (ERK1/2 inhibitor). The results showed that all MAPK are able to inhibit nuclear translocation of C/EBPα, C/EBPβ or C/EBPδ (Figure 3). Likewise, pharmacological inhibition of MAPK resulted in similar down-regulation of high glucose-induced ET-1 gene transcription and mRNA expression (Figure 4). These data suggest that high glucose-activated MAPK-dependent pathways are responsible for both ET-1 induction and C/EBP activation in cultured EC.

### C/EBP isoforms mediate high glucose-induced ET-1 expression

**Figure 5:**
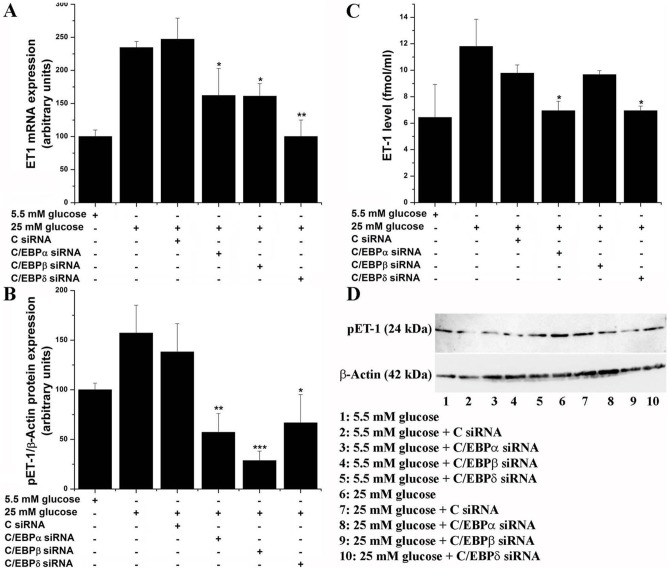
C/EBPs mediate high glucose-induced (A) mRNA expression, (B) pre-pro-ET-1 level, and (C) ET-1 peptide secretion. (D) Representative immunoblots showing the pre-pro-ET-1 protein regulation. Quiescent EC were exposed (24 h) to 5.5 mM or 25 mM glucose in the absence/presence of siRNA sequences directed to silence the expression of various C/EBP subtypes. The ET-1 mRNA and protein expression were assayed by real time PCR, Western blot, and ELISA. n = 4, *P<0.05, **P<0.01. *P*-values were taken in relation to the C siRNA-transfected cells.

Since high glucose activates various members of the C/EBP family, we questioned next the role of various C/EBPs in mediating high glucose-induced upregulation of ET-1. Based on the fact that members of the MAPK activate a large spectrum of transcription factors, including C/EBPs, siRNA-mediated gene silencing was employed to specifically reduce the expression of C/EBPα, C/EBPβ or C/EBPδ isoforms. As depicted in Figure 5A, the high glucose-augmented ET-1 mRNA (≈2.3-fold) was differentially downregulated by siRNA-mediated silencing of the C/EBPα (≈30%), C/EBPβ (≈30%), and C/EBPδ (≈55%). Likewise, silencing of C/EBPα, C/EBPβ or C/EBPδ resulted in a dissimilar decrease of high glucose-induced pre-pro-ET-1 expression level (Figure 5B). Representative immunoblot illustrating pre-pro-ET-1 regulation is shown in Figure 5D.

Comparable results were found on the level of ET-1 peptide secretion into the culture medium. Interestingly, the active ET-1 export seems to be mediated primarily by high glucose activated-C/EBPα and –δ, and not by C/EBPβ (Figure 5C). Nevertheless, the relative silencing efficiency of the C/EBP isoforms and the associated alternative pathways, as well as the multiple levels of intracellular processing implicated in the regulation and maturation of ET-1 in high glucose exposed EC should be considered.

**Figure 6:**
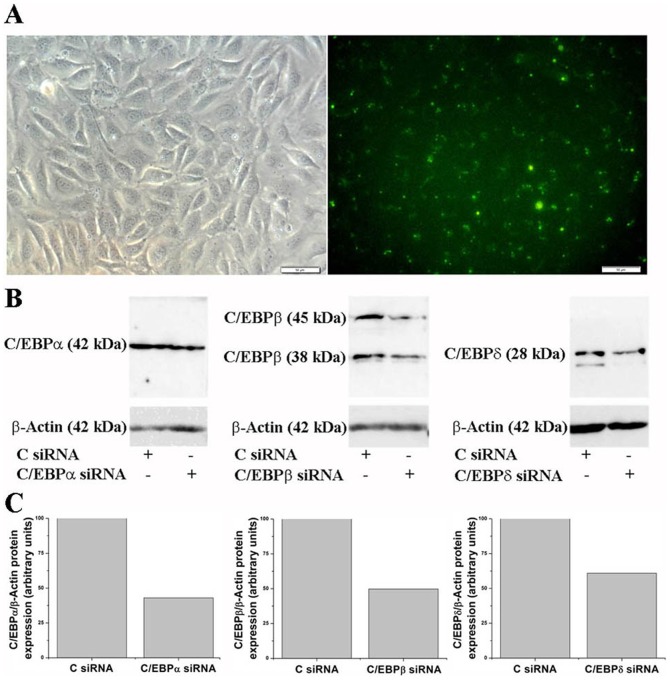
Evaluation of siRNA transfection and silencing efficiency in endothelial cells. Cultured cells were transfected with either control/scrambled (C siRNA), C/EBPα-, C/EBPβ- or C/EBPδ- siRNA. 48-hours after transfection (A) the up-take efficiency of FITC-siRNA was monitored by fluorescence microscopy (x20) and (B, C) the down-regulation of C/EBP protein levels by Western blot. n = 3.

To assess the uptake efficiency of siRNA, control siRNA tagged with FITC was used. The cells were analyzed by fluorescence microscopy (Carl Zeiss, Germany) 48 hours after transfection. A positive FITC reaction was detected in ≈90% of transfected cells. No FITC signal was seen in non-transfected cells. In control experiments, when the transfection was performed in the absence of transfection reagent, FITC-labeled siRNA were not taken up significantly by EC (data not shown). Data about siRNA transfection and C/EBP silencing efficiency are shown in Figure 6.

### Functional analysis of C/EBP elements within the human ET-1 gene promoter

**Figure 7:**
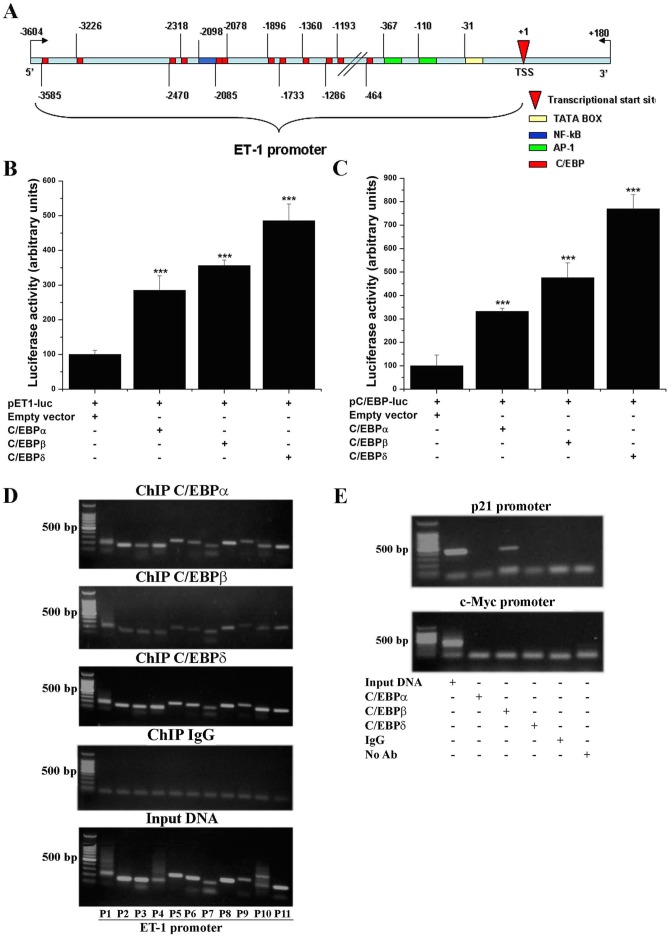
Functional analyses of C/EBP *cis*-acting elements within human ET-1 gene promoter. (A) Schematic depiction of the ET-1 gene promoter and the relative positions of the putative C/EBP binding sites are shown. (B) Analysis of ET-1 gene promoter activity in EC overexpressing C/EBPα, C/EBPβ or C/EBPδ. (C) Validation of C/EBPα, C/EBPβ or C/EBPδ overexpression by using pC/EBP-luc control plasmid. (D) ChIP assessment of C/EBPα-, C/EBPβ- or C/EBPδ – ET-1 promoter interaction in high glucose (25 mM)-exposed EC. IgG was used as negative control to test the specificity of the C/EBP antibodies. Representative agarose gel electrophoresis depicting the predicted amplicon size of the PCR products. (E) ChIP positive control - DNA fragment derived from human p21 gene promoter containing highly conserved C/EBP elements; ChIP negative control - DNA fragment derived from human c-Myc gene promoter that do not contain C/EBP sites. n = 3.

*In silico* analysis (TRANSFAC™, Alibaba2.1) of the human ET-1 gene promoter (≈3600 bp) indicated the existence of 12 highly conserved C/EBP (minimal match of conservation 90%). A schematic representation of the human ET-1 gene promoter depicting the relative positions of the C/EBP sites is illustrated in Figure 7A. To investigate the function of the C/EBP elements in the modulation of ET-1 transcription, cotransfection experiments were done employing pET1-luc construct and C/EBPα, C/EBPβ, and C/EBPδ wild-type expression vectors. The results showed that compared to the control (empty vector) level, a significant up-regulation of the ET-1 gene promoter activity was detected in EC transiently overexpressing C/EBPα (≈3-fold), C/EBPβ (≈3.5-fold) or C/EBPδ (≈5-fold) (Figure 7B). The overexpression of the C/EBP isoforms was confirmed by using pC/EBP-luc control plasmid (Figure 7C).

To further investigate the involvement of C/EBP transcription factors in the regulation of ET-1, chromatin immunoprecipitation were employed to assess the direct interaction of the above indicated C/EBP subtypes with the ET-1 gene promoter. Quiescent EC were exposed for an additional 24 h to 25 mM glucose and the chromatin was processed using polyclonal antibodies against C/EBPα, C/EBPβ or C/EBPδ proteins. As shown by agarose gel electrophoresis analysis, specific interactions among C/EBP proteins and ET-1 gene promoter were detected (Figure 7D). In parallel experiments we amplified from immunoprecipitated chromatin a DNA fragment containing highly conserved C/EBP elements derived from the human p21 gene promoter (positive control) using primers from R&D Systems. In addition, a DNA fragment derived from human c-Myc gene promoter (R&D Systems) that do not contain C/EBP sites served as negative control (Figure 7E).

## Discussion

Dysregulation of the endothelin system play a major role in onset and progression of micro- and macrovascular disorders associated with diabetes mellitus. [14] Produced in excess, ET-1 triggers a series of critical events comprising inflammation, oxidative stress, fibrosis, and cellular phenotypic alterations of the vascular resident cells and infiltrated immune cells which converges to vascular dysfunction and ultimately cardiovascular diseases and downstream complications. [25], [26]

Thus far, the pathophysiological processes involved in the induction of ET-1 are insufficiently elucidated. Several lines of evidence indicate that the vascular inflammation- and growth-promoting transcription factors NF-kB and AP-1 are also important regulators of ET-1 expression. [15], [27] In accordance to this, we have demonstrated previously that the pro-inflammatory signaling pathway, Jak/STAT, mediates the up-regulation of ET-1 biosynthesis in human EC exposed to high glucose concentration. [16]

Compelling evidence highlights that various members of the C/EBP transcription factor family play a major role in the onset and development of cardiovascular diseases. It has been demonstrated that inhibition of C/EBP transcriptional activity by decoy oligodeoxynucleotide reduces the restenosis after angioplasty in hypercholesterolemic rabbits, a process that may be partially mediated by the reduction in ET-1 expression. [28] Moreover, C/EBPβ and –δ have been implicated in the regulation of pre-pro-ET-1 in response to mechanical forces, suggesting that these transcription factors might have a role in modulating vascular tonus by regulating ET-1 synthesis in hypertension. [29] However, there are no data regarding the role of C/EBP family in diabetes and their possible impact on ET-1 expression in EC.

To gain additional insights into the potential regulating mechanisms of ET-1 in diabetes, we have further investigated the role of C/EBP in mediating high glucose-induced up-regulation of ET-1 in cultured human EC. Based on the fact that C/EBPα, -β, and –δ isoforms play a major role in various aspects of vascular pathophysiology, [30]-[32] the implication of these transcription factors in the regulation of ET-1 was considered in this study.

To ascertain the previous findings [16], [33] that high glucose induces ET-1 level and to validate the experimental model, dose-dependent experiments were done in EC exposed to increasing concentrations of glucose. The results proved high glucose augments ET-1 biosynthesis and regulates its expression at multiple levels such gene transcription, mRNA expression, intracellular processing and peptide secretion into the culture medium in both EC line (EAhy926) and in primary cultures of human aortic EC.

To evaluate the effect of high glucose on C/EBP function two different strategies were employed, namely assessment of C/EBP activation by means of nuclear translocation and trans-activation assays employing a C/EBP-luciferase reporter control plasmid. The results showed that high glucose induces C/EBP-associated transcriptional responses. In addition, we found that C/EBPα, -β, and –δ isoforms are differentially activated in response to high glucose.

Members of the MAPK family have been shown to play a role as up-stream regulators of C/EBPs in various cell types. [19], [34], [35] Therefore, to test this hypothesis in EC, various pharmacological inhibitors of the JNK, p38 MAPK and ERK1/2 were used. The results demonstrated that chemical inhibition of each MAPK elicit significant but dissimilar attenuations of the high glucose-induced activation of C/EBPα, -β, and –δ activation, and also ET-1 transcription and mRNA level. These data suggest that high glucose-induced ET-1 is partially mediated by MAPK-triggered C/EBP nuclear mobilization and activation of gene transcription. Still, the above indicated protein kinases are not restricted to the C/EBP family and several other transcription factors (i.e., NF-kB, AP-1) have been demonstrated to be directly or indirectly activated by MAPK.

To overcome this limitation and to increase the specificity, siRNA-mediated gene silencing was used to selectively reduce the expression of C/EBPα, C/EBPβ or C/EBPδ. We have found that silencing of either C/EBPα, C/EBPβ or C/EBPδ reduced significantly the high glucose-induced ET-1 expression.

To further investigate the molecular basis of ET-1 regulation by C/EBP transcription factor family, *in silico* analysis was done to predict the occurrence of the C/EBP *cis*-acting elements within human ET-1 gene promoter. Apart from the typical TATA boxes responsible for the basic promoter activity several putative transcription factor binding sites for NF-kB, AP-1, STAT, Sp1, CREB, and GATA that might influence the ET-1 transcription were found. In addition, 12 highly conserved C/EBP sites were predicted. Activated C/EBP transcription factors bind specific elements RTTGCGYAAY (R: A/G, Y: C/T) in the enhancer or promoter regions to control the transcription of target genes. [18] Nevertheless, specific interactions with less conserved DNA binding sites have been shown. [36]

To test the function of the predicted C/EBP elements within human ET-1 gene promoter, we performed cotransfection experiments using the pGL2 luciferase construct (pET1-luc) carrying the previously characterized ET-1 gene promoter [15] and wild-type expression vectors for C/EBPα, C/EBPβ, and C/EBPδ. Transient overexpression of each C/EBP subtype up-regulated the luciferase activity directed by the ET-1 promoter, a condition that suggests the existence of functional C/EBP binding sites.

To evaluate the physical C/EBP – ET-1 promoter associations in high glucose-exposed EC, ChIP assay was performed using primer sets flanking the putative C/EBP sites. The results showed that C/EBPα, C/EBPβ, and C/EBPδ interact with the ET-1 promoter in the genomic regions displaying the predicted elements. It is noteworthy that in addition to the canonical interactions, several mechanisms of C/EBP-induced gene transcription have been demonstrated including interplays with other transcription factors such as (NF-kB, AP-1, Sp1, CREB) [37]–[40] or recruitment of various transcriptional co-regulators that affect chromatin conformation, a condition that untimely facilitates the trapping of basal transcription factors. [41], [42] Based on that fact that NF-kB and AP-1 are direct regulators of ET-1 transcription, the existence of non-canonical associations among transcriptional complexes formed by heterodimers consisting of C/EBP and other transcription factors that might or might not contain the basic-leucine zipper domain [18] should be considered as well.

Collectively, we assume that a complex interplay among multiple transcription factors, co-activators, and/or co-repressors are coordinately involved in the up-regulation of ET-1 in diabetes and its associated cardiovascular complications.

In this context, the present study brings new conclusive data that in cultured EC exposed to high glucose concentrations the augmented ET-1 level is partially mediated by MAPK – C/EBP signaling pathways. Consequently, pharmacological targeting of the key molecular mechanisms implicated in ET-1 biosynthesis may be used to correct aberrant activated signaling pathways and to improve vascular function in diabetes.

These findings may provide either novel or additional means for the therapeutic management of diabetes-associated vascular diseases. However, the expected impact of these data goes well beyond the diabetes field. Elevated ET-1 is a common occurrence in most cardiovascular pathologies such as atherosclerosis and hypertension. [43] This knowledge may be applicable to all these pathologies since controlling ET-1 level ought to have a beneficial knock-on effect on these diseases.
